# Skin prick tests to multiple pollens and prevalence of IgE specific to profilin

**DOI:** 10.1186/1710-1492-10-S1-A12

**Published:** 2014-03-03

**Authors:** Greg Plunkett, Domingo Barber, Eliseo M Villalobos, Joshua S Jacobs, Jeffrey S Hallett, Tara Mostofi, Agustin Galan Nieto, Tricia Moore

**Affiliations:** 1 ALK-Abelló, Inc, Round Rock, TX, 78664, USA; 2 Institute of Applied Molecular Medicine, CEU San Pablo University, 28003 Madrid, Spain; 3 Allergy Institute of San Antonio, PA, San Antonio, TX, 78231 USA; 4 Allergy and Asthma Medical Group of the Bay Area, Walnut Creek, CA, 94598, USA; 5 Dr. Jeffery Hallett, Round Rock, TX, 78681, USA; 6 ALK-Abelló, E-28037 Madrid, Spain

## Background

Panallergens like profilin are proteins that have very similar sequences and structure. People can develop IgE specific to these highly cross reactive panallergens. The purpose of this study was to determine the prevalence of profilin sensitivity and the contribution to multiple allergen skin test responses. Sensitivity to profilin was established by IgE binding to purified profilins. Possible effects on skin test results were studied by including Queen Palm (QP) allergen in the skin prick testing panel as a possible source of profilin. QP is a species with a low probability of pollen exposure.

## Methods

65 subjects from Central Texas and Northern California were recruited for SPT with Multi-Test®II to a panel of up to 51 allergens. Several extracts were tested for profilin and spanned a range of 0 – 14 microgram/mL. QP containing 10ug/mL profilin was added to the panel as a possible source of panallergen. IgE to various purified component major allergens and profilin was determined using Thermo Fisher ISAC microarray or ADVIA Centaur.

## Results

23 of 65 subjects (35%) had a positive IgE to at least one profilin. Of these, 9 had a >3mm wheal to QP. 20 of 65 subjects (32%), had a positive QP SPT, a similar prevalence of positive SPT as either Timothy grass (43%) or birch (34%) pollen. Subjects reacting by SPT to QP also reacted to multiple allergens, 21 – 100% of allergens tested (average 58%). The multiple allergen SPT sensitivity (percent of 16 allergens) correlated with *in vitro* profilin IgE binding, QP SPT+, sIgE+, 75%; QP SPT+, sIgE-, 56%;QP SPT-, sIgE+, 40%; QP SPT-, sIgE-, 32%.

## Conclusions

Prevalence of IgE to profilin in the current study is similar to reports from European studies. The association of IgE specific to profilin and Queen Palm pollen skin responses suggests this low probability allergen may be useful to identify panallergen responses responsible for multiple sensitivities.

